# Surgical data strengthening in Ethiopia: results of a Kirkpatrick framework evaluation of a data quality intervention

**DOI:** 10.1080/16549716.2020.1855808

**Published:** 2020-12-24

**Authors:** Sehrish Bari, Joseph Incorvia, Katherine R. Iverson, Abebe Bekele, Kaya Garringer, Olivia Ahearn, Laura Drown, Amanu Aragaw Emiru, Daniel Burssa, Samson Workineh, Ephrem Daniel Sheferaw, John G. Meara, Andualem Beyene

**Affiliations:** a Program in Global Surgery and Social Change, Harvard Medical School, Boston, MA, USA; b University of California, Davis Medical Center, Sacramento, CA, USA; c University of Global Health Equity, School of Medicine, Kigali, Rwanda; d College of Medicine and Health Sciences, School of Public Health, Department of Reproductive Health and Population Studies, Bahir Dar University, Bahir Dar, Ethiopia; e Ethiopian Federal Ministry of Health, State Minister's Office, Addis Ababa, Ethiopia; f Jhpiego, Addis Ababa, Ethiopia; g Department of Plastic and Oral Surgery, Boston Children’s Hospital, Boston, MA, USA; h Department of Surgery, Addis Ababa University School of Medicine, Addis Ababa, Ethiopia

**Keywords:** Global surgery, safe surgery, Ethiopia, Kirkpatrick evaluation, monitoring and evaluation in safe surgery

## Abstract

**Background**: One key challenge in improving surgical care in resource-limited settings is the lack of high-quality and informative data. In Ethiopia, the *Safe Surgery 2020* (SS2020) project developed surgical key performance indicators (KPIs) to evaluate surgical care within the country. New data collection methods were developed and piloted in 10 SS2020 intervention hospitals in the Amhara and Tigray regions of Ethiopia.

**Objective**: To assess the feasibility of collecting and reporting new surgical indicators and measure the impact of a surgical Data Quality Intervention (DQI) in rural Ethiopian hospitals.

**Methods**: An 8-week DQI was implemented to roll-out new data collection tools in SS2020 hospitals. The Kirkpatrick Method, a widely used mixed-method evaluation framework for training programs, was used to assess the impact of the DQI. Feedback surveys and focus groups at various timepoints evaluated the impact of the intervention on surgical data quality, the feasibility of a new data collection system, and the potential for national scale-up.

**Results**: Results of the evaluation are largely positive and promising. DQI participants reported knowledge gain, behavior change, and improved surgical data quality, as well as greater teamwork, communication, leadership, and accountability among surgical staff. Barriers remained in collection of high-quality data, such as lack of adequate human resources and electronic data reporting infrastructure.

**Conclusions**: Study results are largely positive and make evident that surgical data capture is feasible in low-resource settings and warrants more investment in global surgery efforts. This type of training and mentorship model can be successful in changing individual behavior and institutional culture regarding surgical data collection and reporting. Use of the Kirkpatrick Framework for evaluation of a surgical DQI is an innovative contribution to literature and can be easily adapted and expanded for use within global surgery.

## Background

One of the challenges in improving global surgical care is the lack of data on the current state of surgical systems, especially in resource-limited settings [1]. In 2015, the Federal Ministry of Health (FMoH) of Ethiopia launched Saving Lives Through Safe Surgery (SaLTS), a 5-year national flagship initiative to build capacity at all levels of the healthcare system to improve access to safe surgical and anesthesia care [2]. One of SaLTS’ key focus areas, *Monitoring, and Evaluation*, is intended to build evidence around the current state of surgery in Ethiopia and the impact of SaLTS implementation [2].

SaLTS, in collaboration with Harvard Medical School’s Program in Global Surgery and Social Change (PGSSC), developed 15 surgical key performance indicators (KPIs) (Table 1) that could feasibly be collected at the facility level and reported nationally. This was one component of the Safe Surgery 2020 (SS2020) program, a multi-stakeholder initiative funded by *GE Foundation* with the primary objective of building surgical capacity in developing countries through implementation of a suite of interventions [2,3]. These indicators are intended to provide a longitudinal and comprehensive overview of national surgical performance. A 16th indicator, surgical referrals out of the hospital, was added to specifically evaluate SS2020 programs.

**Table 1.** Surgical key performance indicators (KPIs) developed by PGSSC and FMoH SaLTS team

Surgical Key Performance Indicators			
1	Surgical Bed Occupancy Rate	9	Protection Against Catastrophic Expenditure
2	Delay for Elective Surgical Admission	10	Surgery, Anesthesia, Obstetrics (SAO) Provider Density
3	Mean Duration of In-Hospital, Pre-Operative Stay	11	Peri-Operative Mortality Rate (POMR)
4	Patient Satisfaction	12	Surgical Volume
5	Rate of First Elective Case On-Time Theater Performance	13	Surgical Site Infection
6	Rate of Cancellation of Elective Surgery	14	Anesthetic Adverse Outcome
7	Blood Unavailability Ratio	15	Rate of Safe Surgery Checklist (SSC) Utilization
8	Emergency Surgical Access	16	Surgical Referrals Out

To assess the feasibility of collecting and reporting these indicators at the facility level, new data collection methods were developed and piloted in 10 SS2020 intervention hospitals in the Amhara and Tigray regions of Ethiopia [4]. These methods expanded on existing collection and reporting mechanisms to ensure a more streamlined implementation process. Existing FMoH surgical patient registries were updated to include all data components needed for calculation of 12 core KPIs (Table 1). Alternate data collection methods were developed for four indicators that were not feasible to capture in these registries. To efficiently roll-out the new tools in pilot hospitals, a Data Quality Intervention (DQI) was implemented and consisted of (1) a comprehensive 3-day training program and (2) 7 weeks of on-site mentoring for all training participants.

This study measures the successes and challenges of the DQI using the Kirkpatrick Method, the most widely utilized and verified mixed-method evaluation tool for training programs [5]. This study is one of the first to use the Kirkpatrick framework to evaluate a training program in a global surgery context and aims to evaluate (1) the impact of the intervention on surgical data quality improvement, (2) the feasibility of a new data collection system, and (3) the potential for national scale-up.

## Methods

### Program design

As part of the SS2020 initiative, the Ethiopian FMoH selected five hospitals in the Amhara region and five hospitals in the Tigray region to receive a number of interventions to increase surgical capacity. These hospitals were used as the pilot sites for the DQI and are evaluated in this study. Site visits were conducted prior to implementation to inform the training and overall design of the intervention. Current data collection and reporting methods were noted in these visits, which informed the development of novel collection methodology.

Led by the PGSSC team, the 8-week DQI included a regional training of local surgical mentors, hospital management, and key surgical staff from each hospital. Participants were trained on the importance of data quality and monitoring and evaluation in surgery, understanding the surgical KPIs, and using new registries for data collection. A subgroup of participants known as KPI focal persons were also trained to enter registry data on REDCap, an electronic data capture platform [6]. Post-training, local mentors and PGSSC team members conducted weekly supportive supervision visits to the hospitals with the goal of collecting 1 month of high-quality patient registry data, which would be used to calculate the KPIs. The hospitals’ progress and experiences were shared at a reporting workshop at the conclusion of the intervention. Additional details about the program and the preliminary KPI data are found in this study’s complementary abstracts on implementation of a surgical data quality improvement intervention [4,7–10].

### Evaluation of the training program

The regional training and its subsequent period of facility-based mentorship were evaluated using the Kirkpatrick model, which is used across many disciplines to measure impact of training courses [5]. Main outcomes were assessed across four levels. Level 1 (reaction) assesses participants’ perceptions on the enjoyment, relevance, and engagement in the training. Level 2 (learning) assesses the degree to which knowledge and skills are acquired and learned. Level 3 (individual behavior) assesses the application of knowledge and/or skills into personal practice. Level 4 (institutional behavior) assessed institutional change and data quality improvements in the hospital. A mixed-methods approach was used to understand the impact of the DQI on these components and guide future improvements.

Kirkpatrick Level 1 assessed the reactions, perceptions, and attitudes of the participants immediately after the initial 3-day DQI training. Individuals were provided with a questionnaire consisting of (1) trainer-centered and learner-centered statements accompanied by a 4-point Likert scale and (2) open-answered questions to provide more feedback and opportunity for participants to reflect on the training.

Level 2 assessed (a) KPI knowledge attainment and (b) subsequent accuracy of implementation of that knowledge. Level 2a consisted of a pre- and post-test of 15 multiple-choice questions covering the training material, administered at the beginning and end of the 3-day training, respectively. Participants were encouraged to indicate their answer and their confidence in the correctness of that answer. Level 2b assessed the overall quality of data captured in the new surgical patient registries and accuracy of electronic data entry. Three registry and REDCap data quality checks were retrospectively conducted immediately post-intervention using registry accuracy, data entry verification, and KPI calculation accuracy.

Level 3 consisted of a 16-item Likert-scale questionnaire administered at the end of the DQI, which assessed the extent of individual behavior change among participants.

Level 4 measured the extent of institutional change and consisted of a 10-item Likert-scale questionnaire administered to all participants, and a focus group at each hospital to discuss the training, data collection and reporting, the intervention as a whole, and other concerns regarding the improvement of the quality of surgical data at their facility. This was performed at the conclusion of the 8-week intervention. All DQI participants were invited, in-person, to attend the focus groups for their respective hospitals. Focus group discussions (FGDs) were semi-structured and included questions designed to understand the primary facilitators and barriers to new learning and behavior change during the DQI. The FGDs were moderated by two male Ethiopian doctoral student researchers trained in qualitative research. PGSSC staff that led the DQI assisted in facilitation of each group since participants did not have prior relationships with the researchers. Researchers assured participants of their external, unbiased roles as evaluators. Each group was conducted over 45 minutes in Amharic and Tigrinya, the local languages of Amhara and Tigray, respectively. Responses were audio-recorded, translated, and transcribed into English for analysis. Field notes were also recorded.

All tools were developed by PGSSC, and informed by existing literature on application of the Kirkpatrick model in evaluations [5,11–14]. Surveys were administered in English, on paper, with interpreters available if needed. All survey and focus group participants were informed of the study components and provided verbal consent to participate. IRB approval was obtained for all activities by both Harvard Medical School and the Ethiopian Public Health Institute.

### Data analysis

This evaluation study used a concurrent embedded approach to mixed-methods analysis [15]. Quantitative tools assessed the degree of knowledge gain by the participants and their perception of behavior change in themselves and the surgical team, while the qualitative tools allowed more in-depth exploration of the mechanisms for change and the extent of impact.

Level 1 results were analyzed quantitatively by calculating median responses to Likert questions. Open-ended questions were recorded and saved to inform further iterations of the training design.

For level 2a, test score, confidence score, and confidence indicator were all calculated pre- and post-test to determine knowledge improvement during the regional training component of the DQI. First, knowledge test scores were calculated on a percentage scale indicating the percentage correct. Second, a confidence score was developed based on Gardner-Medwin confidence-based assessments [12]. Responses were weighted based on whether the answer was correct and the participant-indicated degree of confidence. Respondents were able to indicate Low Confidence (C = 1), Medium Confidence (C = 2), and High Confidence (C = 3). This methodology rewards high confidence, correct answers and punishes high confidence, incorrect answers. Finally, dividing the confidence score by the test score provides the value of the confidence indicator, which measures the degree to which participants are confident in their understanding of the subject material. A value of 1.00 to 1.50 shows high confidence and understanding, while values less than 1.00 indicate over- or under-confidence in the participant’s understanding of the knowledge. Over-confidence results from many high confidence, wrong answers; under-confidence results from many low confidence, correct answers. The lower the confidence indicator, the more overconfident a participant is in their knowledge.

Level 2b data quality checks were analyzed using a three pronged-approach: (1) *Cross-Registry Consistency* in data components found across multiple registries was assessed with enumeration of inconsistencies in a 10% random sample of patient cases, (2) *Data Entry Verification* was conducted by reviewing a random sample of 10% of patients to tally errors in data entry of paper registries, and (3) *Calculation Accuracy* was evaluated by determining percent error between 5 KPI values calculated from REDCap data (the measured value) and values calculated directly from registries (the accepted value from source data).

Individual and institutional behavior change were assessed in Levels 3 and 4. Likert scale responses from questionnaires were tabulated in Excel and the median response was measured for each item. Behavior change was also measured by FGDs in Level 4 and analysis consisted of multiple steps that follow standard qualitative analysis methodologies, primarily focusing on grounded theory [16,17]. Five authors coded each transcript independently to generate preliminary thematic codes and identify representative quotes. After a series of discussions, the team consolidated and summarized emerging themes when consensus and saturation were reached.

## Results

### Training reaction (level 1)

From February to March 2018, 34 personnel from 5 hospitals attended the Amhara regional training, while 38 personnel participated across 5 hospitals from April to May 2018 in Tigray. All 72 attendees were enrolled in the Kirkpatrick study with verbal assent. Of these attendees, approximately 66% were male and 34% were female. About 66% reported having clinical roles in their respective hospitals, while 23% reported having an administrative or data management role. The remaining 11% were in leadership roles with clinical backgrounds. Overall, the reaction to the training was positive, with the majority of participants either ‘agreeing’ or ‘strongly agreeing’ with the largely positive, trainer- and learner-centered statements provided on reaction surveys [13]. The median response for each of the seven statements was 4, indicating that the participants strongly agreed that the training was enjoyable, helpful, valuable, and increased understanding and confidence in the participants about collection and reporting of the surgical key performance indicators.

### Knowledge gain (level 2)

#### Testing & confidence

**Figure 1.**
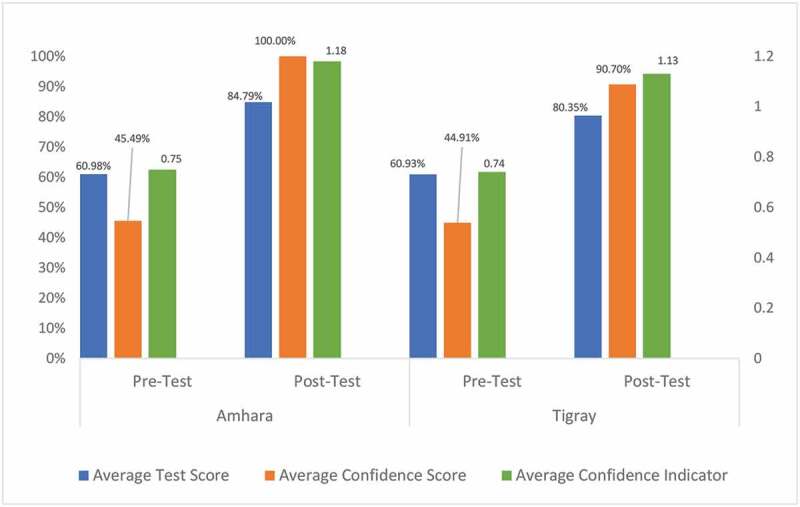
Amhara and Tigray pre- and post-test scores comparison (Kirkpatrick level 2)

There was an overall increase in participants’ test scores, confidence scores, and confidence indicators from pre-test to post-test (Figure 1). Average test score increased by 23.81% in Amhara and 19.42% in Tigray. Average confidence score increased by 54.51% in Amhara and 45.79% in Tigray. Average confident indicator increased by 0.43 points in Amhara (0.75 to 1.18) and 0.39 points in Tigray (0.74 to 1.13). Both confident indicator improvements represent changes in participants’ confidence in correct answers. At pre-test, 37 of the 72 (51%) participants across regions were overconfident in their answers, while at post-test 16 of 72 (22%) participants were overconfident.

#### Registry & data entry accuracy

The results of the data quality assessments are as follows:

(1) *Cross-Registry Consistency*: A manual review of 932 data fields across 85 randomly selected patient cases in the 10 intervention hospitals found minimal inconsistencies across registries (7.6%); (2) *Data Entry Verification*: An assessment of the same 85 patient cases in REDCap identified data entry errors from the registries to REDCap in 14.7% of all data fields reviewed; (3) *Calculation Accuracy*: The percent error between KPIs calculated using the patient registries (source data) and REDCap data were greater than 5% for 17 of 50 compared values (Table 2).

**Table 2.** Key performance indicator (KPI) calculation comparison, REDCap v. registry (Kirkpatrick level 2)

Percent Difference Between Calculated Values of Key Performance Indicators (KPIs) (REDCap v. Registry)					
	*Difference (%)*				
*Hospital*	Anesthetic Adverse Outcome	Post-operative Mortality Rate	Surgical Site Infection	Surgical Volume	Referrals Out
1	0.00%	0.00%	0.00%	**4.10%**	**5.40%**
2	0.00%	0.00%	0.00%	0.00%	**9.10%**
3	0.00%	0.00%	0.00%	**33.30%**	**38.00%**
4	0.00%	0.00%	**100.00%**	**22.00%**	**12.80%**
5	**100.00%**	0.00%	0.00%	**7.70%**	**100.00%**
6	0.00%	0.00%	**100.00%**	2.94%	**76.09%**
7	0.00%	0.00%	0.00%	2.78%	**2.44%**
8	**100.00%**	0.00%	0.00%	3.57%	0.00%
9	0.00%	0.00%	0.00%	**6.00%**	**38.89%**
10	0.00%	0.00%	**100.00%**	0.00%	**100.00%**

### Behavioral change (levels 3–4)

#### Quantitative results

**Figure 2.**
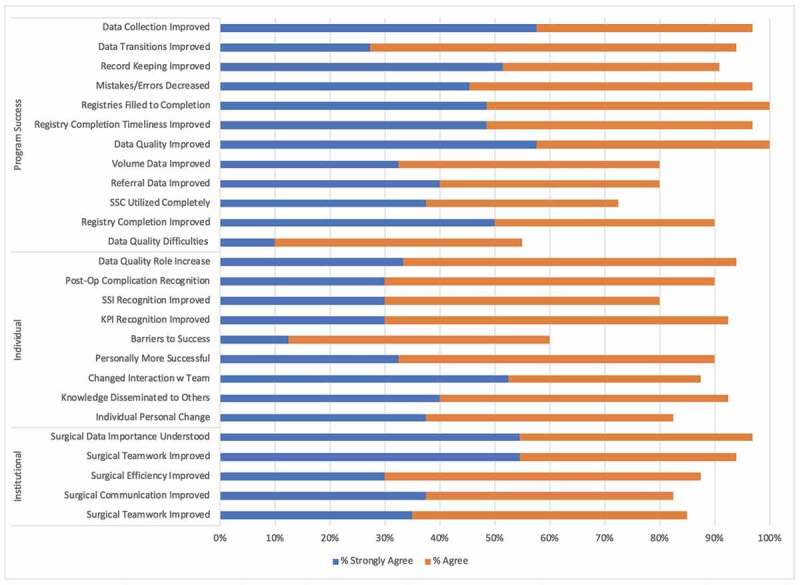
Percentage of participants who agree and strongly agree with program success, individual behavior change, and institutional behavior change items following the intervention (Kirkpatrick levels 3 & 4)

On the Level 3 and 4 questionnaires, individual behavior change was measured by nine items and institutional behavior change was measured by 5. Median response for both sets of items was 3. On average, 84% of participants ‘agreed’ or ‘strongly agreed’ that individual behavior changes were observed after attending the training, while, on average 93%, agreed that institutional behaviors were observed (Figure 2).

Twelve items on the questionnaires were designed to measure program-related change (Figure 2). These focused on data quality change, implementation of the data collection system, and how understanding of the indicators improved. Median response for program success items was 3. On average, 92% of participants ‘agreed’ or ‘strongly agreed’ that program successes were observed after attending the training.

#### Qualitative results

Of the 72 participants that assented to this study, 64 (89%) participated in the FGDs. Ten focus groups were implemented, one in each intervention hospital; approximately four to eight participants attended each FGD. Non-response was attributed to lack of availability. Examples of behavior change emerged in FGDs. Participants shared how the intervention changed their roles and perspectives on data. Five prominent themes emerged that provide a positive overview of the effect of the training program and subsequent mentorship on intervention hospitals (Table 3). Participants identified areas of improvement and three primary challenges to intervention implementation (Table 4).

**Table 3.** Qualitative impact of training as perceived by participants, grouped by theme (Kirkpatrick level 4)

Theme	Description	Quotes
*KPI Knowledge Gain and Data Utilization*	Many participants expressed increased knowledge gain regarding the surgical KPIs and how the hospital utilizes data better because of the intervention.	“The introduction of KPIs has improved the quality of care. Before the training, we were reporting [data] but it was lacking completeness. Besides, there was no calculation of the KPIs. However, after the training, we calculated the KPIs and understood its benefits. This provides us an opportunity to measure the indicators and monitor our progress objectively. We can now determine the safety checklist [use], the mortality related to the service, SSI and the like. Before the training, there was no measurement of the pre-operative care, safety checklist and even blood availability. The calculation of blood availability would help us to identify the number of referral outs associated with blood availability. – Tigray, Hospital 2 There has been improvement in acknowledging the value of data since the training. The training also makes people focus on very key performance indicators that are important in monitoring and evaluating the activities. – Amhara, Hospital 2 We also became aware of the advantages of properly managed data … such as for planning, research and decision – Amhara, Hospital 1 … the intervention helped us to identify our gaps and knowing the gaps leads us to strive towards the solution.” – Amhara, Hospital 2
*Agency*	Leadership and accountability growth in staff were reported by many, especially regarding an increase in confidence or agency around data and their professional role because of the training.	“The training has brought remarkable changes on the following activities … it has created sense of ownership and accountability among staff members – Amhara, Hospital 2 I and my colleagues perceived that we are responsible for the success and failure of data quality in this hospital. With this in mind, we have improved in data documentation after training. – Amhara, Hospital 3 Even recording and filling up of the data was based on the level of willingness of the professionals: some may fill while some others may not. But now, all staffs are responsible to record the data of what they did.” – Tigray, Hospital 1
*Teamwork and Communication*	Over three-quarters of the hospitals reported that teamwork and communication within the surgical team and the hospital improved because of the intervention.	“Thus, the training comes with the concept that safe surgery is the role of all staff in the hospital and should be approached as a team. What makes a hospital is when the hospital works [as a] team … the team work and team sprit has improved.” – Tigray, Hospital 5 “Surgical Data quality has been increasing by using standardized registries and well oriented staff. Lessons are also obtained and we are trying to scale up this documentation system to non- surgical units as well.” – Amhara, Hospital 4 “Regarding the teamwork, the SaLTs members consist of staff from higher level personnel of the hospital to the bottom level, which gives strength and attention to the issues. Especially, the CEO is a member of the team and he raises it as an agenda always, which keeps the issue closer [for] follow up.” – Tigray, Hospital 4 “Health is team work and everybody from the cleaner to the higher officials is involved in patient management even if the role and depth of involvement varies.” – Amhara, Hospital 1
*Data Quality*	Data quality improvements were cited by all hospitals, indicating that the quality of data in their hospital was positively impacted by the program	“There is also much change in the data quality. There was a weakness and carelessness to record what is done but now it has changed. After[ward] the training staff became curious [about] recording what they did.” – Tigray, Hospital 5 “Data quality has been improved and the following have made their own contribution in this aspect: One, things are critically evaluated periodically by team members and corrective actions are being taken accordingly.” – Amhara, Hospital 2 I can say that data quality in this hospital has improved well. For example, surgical site infection, which did not have ownership before the training, got its own registry and responsible person after the training. Moreover, anesthetic complications, referral system, recording and reporting of canceled surgical cases were all improved after the training.” – Amhara, Hospital 1
*Surgical Services*	Respondents indicated that the training and knowledge gained have allowed for more discussions around improving quality of surgical services and efficiency of hospital administration	“One component of the training was surgical site infection. In our context, the attention given to the issues was limited. But, after the training we are giving attention to it through training. We are looking at the reasons that cause the infection and training for personnel regarding the issues is provided accordingly.” – Tigray, Hospital 1 “Data collection and reporting has become uniform across all units of the hospital. Until the training we were recording data using different formats. We also have started processing, interpreting and utilizing data for our consumption.” – Amhara, Hospital 2 “ … the intervention helped us to identify our gaps and knowing the gaps leads us to strive towards the solution.”- Tigray, Hospital 1

**Table 4.** Reported challenges to implementation, grouped by theme (Kirkpatrick level 4)

Theme	Description	Quotes
*Buy-In Issues*	There were some difficulties in the beginning of the intervention, as reported by some facilities, with getting buy-in from providers for the new registry system, especially those that did not attend the training.	“ … initially there were attitude problems among some staff members in anesthesia department. As you know timing of registration is critical for patients. In this aspect, there are checklists to be filled before, during and after operation.” – Amhara, Hospital 1
*Difficulties Collecting Data*	All hospitals reported difficulties collecting data. These issues ranged from lack of knowledge from members who didn’t attend trainings and issues with human resources.	“As a data collector, it was not an easy task in registering, compiling and online reporting of data using RED Cap software from seven registries weekly. I was using extra time- lunch, night and week end- especially during the first two to three weeks after the training” – Amhara, Hospital 4 “Though the final compiled activities are reported using electronics, still data are recorded manually using hard copies. However, if the registry had been computerized, it could be possible to save time, reduce errors, and retrieve data easily. Thus, there needs to be computer access in each unit to register activities routinely. Fortunately, this hospital has wireless internet access, and if computers are availing in each department it is possible to give comprehensive services.” – Amhara, Hospital 1 “ … we have no separated surgical ward. Medical ward and surgical ward are both in one. Similarly, all the patients admitted in the GYN [ward] may also be surgical cases. Therefore, the SaLTs team should be further expanded to improve the reach of the intervention.” Tigray, Hospital 1
*Continued Training and Follow-Up*	Some hospitals described the need for continued training and follow-up in order to reassess data quality and provide refresher trainings to combat turnover rates in the staff.	“There are also challenges related to data recording and reporting because not all staff are trained and there is also a high turnover of the trained staff. I am observing lack of ownership among non-trained staff … especially among nurses. Therefore, unless mechanisms are designed for continuous onsite training, there might be interruption on data quality management.” – Amhara, Hospital 3 “I suggest regular mentoring, at least every three months, for timely identification of the strong and weak parts of our activities” – Amhara, Hospital 1 “There should also be regular review meetings to look at progress to prevent relapse to the old system” – Tigray, Hospital 1

### Training successes

#### Knowledge gain and data usage

The trainees’ understanding of the necessity of data and consequences of inaccurate or missing data was stressed among all hospitals. Respondents highlighted core KPIs that they believed to be especially necessary, including Safe Surgery Checklist (SSC) utilization, surgical referrals out, surgical volume, and anesthesia complications (Table 1). On-site mentoring was highlighted as a significant reason for the improved data knowledge. The hospitals developed quality improvement projects with a better understanding of the indicators and their importance in ‘planning, research, and decision [making]’ regarding surgical care.

#### Increased sense of agency

Participants expressed improved confidence in and understanding of their role, including expanded responsibilities for data collection and reporting. Most respondents agreed that a sense of ownership and/or accountability of the data management systems increased in their hospitals due to the greater clarity of data ownership roles following the trainings.

#### Improved teamwork and communication

The training provided opportunity for greater collaboration among surgical teams to collect and report data. Many respondents noted improvement to the workflow within surgical teams, with everyone doing their part to record and report data. Some respondents noted that training objectives were embraced by hospital management. One hospital reported that the learnings were applied to other non-surgical units. Teamwork and communication were stressed as being crucial to the successful implementation of an improved data management system.

#### Improved data management systems and data quality

Translating the trainings into practice is a critical component of the intervention. Respondents almost unanimously agreed that the new registries better captured KPIs than the old data system, especially the new Surgical Site Infection Logbook. With clarity on data ownership established during the trainings, the process of recording and reporting data was simplified and facilitated the input of more complete and accurate data.

#### Motivation to improve quality of surgical services

A long-term goal of this intervention was to affect change in the patient safety culture, utilizing the new understanding of data and collection mechanisms to inform safer practices. Participants spoke about how surgical safety is important but had not been a priority prior to this intervention. The DQI helped them better understand how data informs better surgical services and motivated them to apply KPIs to improve services and processes.

### Challenges to implementation

The primary challenge to implementation immediately post-training was ***lack of staff buy-in***. While many key staff at each hospital were present during training, other integral staff to the data management process were absent. Training participants experienced difficulties in receiving ‘second-hand’ support from these staff. Attempting to shift the culture around data for non-training staff proved to be difficult, but, ultimately, successful.

***Lack of human resources*** to effectively implement training objectives in the long term was another challenge. New responsibilities for existing staff under the new data management system led to additional work. Respondents suggested automation of data entry and reporting to combat the lack of human resources while also acknowledging that the lack of computers and reliable internet connectivity were major impediments. There were specific ***complaints about the format of the new registries***, which were not customized to each facility. Some hospitals would have preferred separate OR registries for the obstetrics units and surgical units; others felt that the registries were missing important data fields.

Due to frequent turnover of staff, respondents identified the ***need for additional training and on-site mentorship*** to build institutional knowledge of the KPIs and the new data management systems. Regular ‘review’ meetings were noted as a means to prevent relapse to the old system. Some respondents requested periodic training refreshers for ‘timely identification of the strong and weak parts of our activities,’ as they were concerned that ‘unless mechanisms are designed for continuous onsite training, there might be interruption on data quality management.’

## Discussion

### Interpretation of results

This paper describes the evaluation of a DQI for the collection and reporting of surgical KPIs in Ethiopia using the Kirkpatrick model. Participants agreed that the DQI effectively met its objectives. Knowledge and confidence improved pre to post-testing. Data quality checks identified areas of improvement in data captured, yet discrepancies and mistakes were seen in fewer than 15% of cases. Qualitatively, participants agreed that there were impactful changes in individual behavior and institutional culture.

Each evaluation tier provided insight into key areas of impact. Level 1 training reaction results showed improved knowledge of the KPIs, increased comfort with the new registries, and better understanding of the data collection methods. Improvement in participant test scores, confidence scores, and confidence indicators in Level 2a reflect the increase in understanding of the aforementioned components. A post-test confidence indicator greater than 1.00 in both regions indicates all participants had a good grasp of what information they know versus information they do not. To our knowledge, this is the first time that Gardner-Medwin confidence scores and indicators have been included in the Kirkpatrick Evaluation [14].

Level 2b revealed quality issues in data collection and entry. The input of data in the paper-based surgical registries had minimal inconsistencies across registries and few missing fields. However, verification of data entered into REDCap shows discrepancies from the paper-based source data. The electronic data entry errors reflect a misunderstanding of REDCap, a new tool to all participants. Lack of adequate staffing to implement this task may have contributed to the mistakes identified. This shows the difficulty of implementing an accurate and reliable registry and data entry system in a low-resource setting [6].

Mixed-method analysis in Levels 3 and 4 revealed that participants agreed that both individual behavior and team behavior changed because of the intervention. The DQI was perceived to be successful in improving data quality, indicator understanding, and data collection system use. The main FGD themes (Table 3) indicate that this program can be effective in providing the knowledge and agency that individuals and teams need to successfully implement a DQI. The combination of intensive training and supportive supervision created an environment in which participants felt comfortable and knowledgeable on the indicators, the use of the new registries, and the data collection [18].

The challenges to implementation highlight difficulties in changing data culture within a hospital. Main challenges resulted from lack of buy-in, lack of human resources, and need for additional training and mentorship. Staff turnover also affected implementation. Individuals on-boarded after the training did not feel knowledgeable about the DQI. More emphasis on human resource pipelines and additional trainings and mentorship could alleviate these issues. Modifying the intervention specifically to each hospital setting also could lead to higher uptake and success.

### Results in the context of literature

While the Kirkpatrick Model has been used to assess a training for the WHO SSC, this is the first instance it has been used to evaluate a DQI in global surgery [19]. Previous mentoring training programs in Sub-Saharan Africa using Kirkpatrick agree that mentoring is critical to the implementation of training programs in global surgery [19–21].

Improved knowledge is consistent with other trainings evaluated using the Kirkpatrick method in low-and-middle income countries (LMICs). Dorri and colleagues found favorable reaction, knowledge gain, and behavior change among a CPR in-service training in Iran [22]. A study in Laos found positive results of continuing professional development training among providers [23].

FGDs revealed that buy-in from local, regional, and national partners was important to program success. These findings are consistent with other studies that found that scale-up is dependent on active partners, mentorship, and collaboration [20,21] Participants also reflected on the benefit of mentors’ visits. In order to maintain high-quality data systems, literature supports that routine audits and mentoring need to continue to ensure sustainability [24,25].

### Implications and recommendations

The results of this study are largely positive and make evident that surgical data capture is feasible in low-resource settings and warrants more investment in the field of global surgery. This type of training and mentorship model can be successful in changing individual behavior and institutional culture regarding surgical data collection and reporting. The use of the Kirkpatrick Framework for evaluation of a surgical DQI is an innovative contribution to the literature and can be easily adapted and expanded for use within global surgery.

This study has provided sufficient evidence to warrant further exploration of scale-up of surgery DQIs in Ethiopia and other LMICs. To further this end and ensure sustainability of positive changes seen in intervention hospitals, the Ethiopian FMoH and collaborating partners should be encouraged to take ownership of the program. We recommend a two-pronged approach to adaptation and scale-up at a national level: (1) scaling the surgical registries that were piloted for this intervention across all hospitals providing surgical care in the country, and (2) implementing a similar DQI to accompany a national rollout of the aforementioned registries. Because our DQI was resource-intensive, significant adaptation of the latter component may be necessary to accommodate national implementation.

### Limitations

Our evaluation has limitations. Since the KPI knowledge post-test was only administered immediately following the regional training, knowledge retention was not measured. Surveys were conducted in English with translation available upon request, which may have been a barrier for non-English speaking respondents who did not make this request.

Since most data are self-reported, responder bias may exist. Bias from participants’ fear of speaking critically about colleagues or hospital systems may arise as well. The study subjects were limited to training participants; perspectives of non-participants affected by the intervention were not assessed.

Only primary and general hospitals participated in the intervention, so lessons learned cannot be generalized to regional and national hospitals. Despite these limitations, our mixed-methods evaluation design remains rigorous and holistically captures the impact the training made on the participants and the hospitals.

## Conclusion

The results of this DQI provide insights into a few key areas of impact: a measurable gain in knowledge and understanding of surgical data; mixed results about the efficiency of data quality and electronic entry; increased confidence on the subject; general consensus on positive individual behavior change through proactive engagement in data collection and reporting; and noticeable impact on institutional culture around data integrity and its role in decision-making. To ensure sustained impact of the program and to potentially motivate national scale-up, further alignment of our efforts with Ethiopian stakeholders will be crucial. It will be important to act quickly to build on the momentum of the program and the global surgery movement.

## Paper context

The availability of high-quality surgical data is an ongoing challenge in low-resource settings. An innovative Data Quality Intervention (DQI) was implemented in rural Ethiopia. Evaluation results were positive and make evident that surgical data capture is feasible in low-resource settings and warrants more investment in global surgery efforts. This type of training and mentorship model can be successful in changing individual behavior and institutional culture regarding surgical data collection and reporting and should be scaled.
